# Five-year outcomes of EVO implantable collamer lens implantation for the correction of high myopia and super high myopia

**DOI:** 10.1186/s40662-021-00264-0

**Published:** 2021-11-09

**Authors:** Xun Chen, Xuanqi Wang, Yilin Xu, Mingrui Cheng, Tian Han, LingLing Niu, Xiaoying Wang, Xingtao Zhou

**Affiliations:** 1 Fudan University Eye Ear Nose and Throat Hospital, No. 19 BaoQing Road, XuHui District, Shanghai, 200031 China; 2 National Health Commission Key Lab of Myopia (Fudan University), Shanghai, China; 3 Shanghai Research Center of Ophthalmology and Optometry, Shanghai, China

**Keywords:** Posterior chamber phakic intraocular lens implantation, High myopia, Super high myopia, EVO ICL

## Abstract

**Background:**

To evaluate the long-term safety, efficacy, predictability, and stability of implantable collamer lens with a central hole (EVO ICL) implantation for correcting high myopia (HM) and super high myopia (SHM).

**Methods:**

This prospective study evaluated 83 eyes of 46 patients who were divided into groups based on their spherical equivalent refractive error (SE): HM group (− 12 D ≤ SE < − 6 D) and SHM group (SE < − 12 D). They were followed up for 5 years after ICL implantation; assessments of uncorrected distance visual acuity (UDVA), corrected distance visual acuity (CDVA), manifest refractive error, axial length, intraocular pressure, corneal endothelial cell density, and vault were conducted, and a questionnaire was administered.

**ResuIts:**

At 5 years postoperatively, the safety indices of the HM and SHM groups were 1.03 ± 0.10 and 1.32 ± 0.39, and the efficacy indices were 0.83 ± 0.25 and 0.86 ± 0.32, respectively. In the HM group, 60.47% and 79.07% of the eyes were within ± 0.50 D and ± 1.00 D of the attempted correction, while it was achieved for 22.50% and 47.50% of the eyes in the SHM group, respectively. The SE of the HM group decreased from  − 9.72 ± 1.41 D preoperatively to 0.04 ± 0.39 D 1 month postoperatively and − 0.67 ± 0.57 D 5 years postoperatively, while in the SHM group, it decreased from − 15.78 ± 3.06 D preoperatively to  − 0.69 ± 0.97 D 1 month postoperatively and − 1.74 ± 1.19 D 5 years postoperatively.

**Conclusion:**

EVO ICL implantation is safe, effective, and predictable for correcting HM and SHM. CDVA improved more after surgery for SHM, but the growth of axial length still needs attention.

## Background

China is a large country with a high prevalence of myopia, and the degree of myopia is generally severe. The proportion of patients with high myopia (HM) and super high myopia (SHM) is higher than that in Western countries [1, 2]. Many doctors and patients are concerned about the correction of low to moderate myopia, either by corneal refractive surgery or implantable collamer lens (ICL) implantation, which has been shown to achieve good visual and refractive results [3–5]. Much attention should be given to patients with HM and SHM because they lack self-confidence, and their choice of professions is limited. Therefore, surgery allows both a correction of the refractive defect and a significant change in personality, career, and lifestyle.

In the correction of HM using ICL implantation, the cornea and its biomechanics are not affected. The correction range is wide and does not involve limiting the corneal thickness; therefore, it has been widely used in clinical practice [5–7]. Since the advent of clinical application of EVO ICL, several short-term studies [8–11] have confirmed its safety, efficacy, predictability, and stability in correcting HM. However, doctors and patients are concerned about its long-term safety, efficacy, and stability. To the best of our knowledge, there are no reports on the long-term comparisons of EVO ICL implantation used for the correction of HM and SHM. SHM is often accompanied by posterior staphyloma, elongation in axial length, and the presence of myopic maculopathy [12, 13]. Therefore, this study is based on the hypothesis that the postoperative corrected distance visual acuity (CDVA) improvement space of SHM may be larger than HM because of the poor CDVA preoperatively and that the postoperative axial elongation may be easier. Therefore, this study investigated the safety, efficacy, predictability, and stability of EVO ICL implantation for the correction of HM and SHM for 5 years after surgery to objectively evaluate its long-term visual and refractive outcomes of EVO ICL implantation in the correction of SHM.

## Patients and methods

### Patients

This study adhered to the tenets of the Declaration of Helsinki and was approved by the Ethical Committee Review Board of Fudan University Eye and ENT Hospital. All patients provided written informed consent after the possible risks and benefits of the study were explained.

Patients aged 20–40 years with preoperative spherical equivalent (SE) of − 6.00 D or higher were included in this study. Complications occurring after the surgery, failure to understand the risks of surgery or have unrealistic expectations of surgery outcomes, corneal degeneration, or endothelial cell density < 2000 cells/mm^2^, anterior chamber depth < 2.8 mm, refractive media opacity that severely disturbed vision, a history of autoimmune diseases, and a history of ocular diseases (uveitis, cataract, glaucoma, or retinal detachment) other than myopia and astigmatism were regarded as exclusion criteria.

### Pre- and postoperative protocol

Patients were followed up for five years. Assessments of uncorrected distance visual acuity (UDVA), CDVA, manifest refractive error, axial length (IOL Master, Carl Zeiss, Germany), intraocular pressure (IOP, Tonemeterx-10, Canon, Japan), corneal endothelial cell density (SP-3000P, Topcon Corporation, Japan), and vaults (Pentacam HR, Type 70900; Oculus Optikgeräte GmbH, Wetzlar, Germany) were conducted. A questionnaire with the following questions was also administered:

1. “Does ICL implantation change your self-image, make you feel more beautiful or more handsome?”
2. “Have you become more confident after ICL implantation?”
3. “Did ICL implantation help you in your career?”
4. “Is life more convenient after ICL implantation?”
5. “If there is another choice, will you choose the ICL implantation again?”
6. “Are you satisfied with ICL implantation as a whole?”

### EVO ICL

The EVO ICL (STAAR Surgical, Switzerland) is a plate-haptic single-piece intraocular lens made of collamer. It has a central convex-concave optical zone and incorporates a forward vault to minimize contact with the crystalline lens. A 360 μm central hole was included to improve aqueous humor circulation, eliminating the need for preoperative laser peripheral iridotomy. The EVO ICL corrects − 0.50 to − 18.00 D myopic spherical refraction and up to − 5.00 D cylindrical refraction. There are four sizes: 12.1 mm, 12.6 mm, 13.2 mm, and 13.7 mm. Power calculation of the EVO ICL was performed by the manufacturer using a modified vertex formula, according to the provided preoperative refractive parameters. The size of the implanted EVO ICL was determined based on the white-to-white horizontal corneal diameter and anterior chamber depth. [14, 15].

### Surgical procedure

All surgeries were performed by experienced surgeons (XW and XZ). The implantation of ICL and the surgical procedures were the same as those used in our previous studies [14, 15]. During the surgery, a 3-mm temporal corneal incision was made at the temporal corneoscleral limbus. Then, an EVO ICL was inserted into the anterior chamber with an injector cartridge after a viscoelastic surgical agent (1.7% sodium hyaluronate; Bausch & Lomb, China) was injected into the anterior chamber to maintain the anterior chamber depth. Sometimes, an additional viscoelastic agent was then placed on the top of the ICL, and an ICL positioning instrument was used to sweep the four haptics of the ICL beneath the iris. Subsequently, a balanced salt solution was used to irrigate the viscoelastic agent. Finally, the surgeon gently pressed the cornea with a cotton swab to close the incision.

### Statistical analysis

This study analyzed six outcomes: (1) change in lines of CDVA from preoperative to postoperative measurements (safety); (2) postoperative UDVA compared to preoperative CDVA (efficacy); (3) attempted versus achieved spherical equivalent correction (predictability); (4) changes of SE and axial length; (5) intraocular pressure, vault, and endothelial cell density; (6) questionnaire results.

Statistical analyses were performed using SPSS (version 20.0; SPSS Inc., IBM, USA), and the results are expressed as mean ± standard deviation (range) values. Kolmogorov–Smirnov test to verify the assumption of normality for the statistical analyses. For continuous variables, summary statistics were analyzed using repeated measures analysis of variance with Bonferroni-adjusted post hoc comparisons and student’s t tests as appropriate. For categorical variables, summary statistics were analyzed using chi-square tests. Two-tailed hypothesis testing was performed, and statistical significance was set at *P* < 0.05.

## Results

### Patient demographics

A total of 83 eyes of 46 patients (12 men and 34 women) who underwent EVO ICL implantation were included in this study. Their mean age was 28.47 ± 5.86 (20 to 40) years. The mean preoperative SE was − 12.64 ± 3.84 (− 6.12 to − 24.50) D. According to the preoperative SE, the patients’ eyes were divided into two groups: HM group (− 12 D ≤ SE < − 6 D), including 43 eyes (24 patients) and SHM group (SE < − 12 D), which included 40 eyes (22 patients). Preoperative data for the two groups are listed in Table 1.

**Table 1:** Distribution of preoperative characteristics

Parameter	HM group	SHM group	*P* value
N, eyes	43	40	
Age (years)	27.92 ± 6.09	28.82 ± 5.61	0.61
Gender (male:female)	5:19	7:15	0.40
UDVA (logMAR)	1.46 ± 0.42	1.78 ± 0.34	< 0.001
CDVA (logMAR)	− 0.02 ± 0.04	0.12 ± 0.14	< 0.001
Refractive errors (D)			
Spherical	− 8.94 ± 1.34	− 14.83 ± 2.90	< 0.001
Cylindrical	− 1.56 ± 1.12	− 1.90 ± 0.91	0.13
Spherical equivalent	− 9.72 ± 1.41	− 15.78 ± 3.06	< 0.001
Keratometric value (D)			
Flat K	43.14 ± 1.44	42.80 ± 1.47	0.30
Steep K	44.69 ± 1.50	44.30 ± 1.60	0.26
WTW diameter (mm)	11.90 ± 0.44	11.92 ± 0.40	0.83
IOP (mmHg)	14.27 ± 2.99	15.36 ± 3.04	0.10
CCT (mm)	521.35 ± 29.55	526.15 ± 31.24	0.48
Axial length (mm)	27.08 ± 0.99	29.68 ± 1.64	< 0.001
ECD (cells/mm^2^)	2689.56 ± 286.37	2773.89 ± 206.10	0.14
ICL size (mm)	13.06 ± 0.52	13.12 ± 0.46	0.57

*HM *high myopia; *SHM *super high myopia; *N* number of eyes; *UDVA* uncorrected distance visual acuity; *CDVA* corrected distance visual acuity; *D* diopters; *K* keratometry; *STS* sulcus to sulcus; *IOP* intraocular pressure; *WTW* horizontal white-to-white diameter; *ACD* anterior chamber depth; *CCT* central corneal thickness; *ECD* corneal endothelial cell density; *ICL* implantable collamer lens

### Safety

No complications were found during the surgery and pupillary block, pigment dispersion, uveitis, glaucoma, cataract, and endothelial cell loss > 30% were not observed. Macular hemorrhage occurred in one eye and the CDVA recovered after pharmaceutical treatment. Mild anterior subcapsular opacifications were observed in three eyes, which were still under follow-up observation. These cases did not affect the results and statistical analyses. To avoid interference with this study, these cases were excluded when calculating the refractive results.

**Fig. 1:**
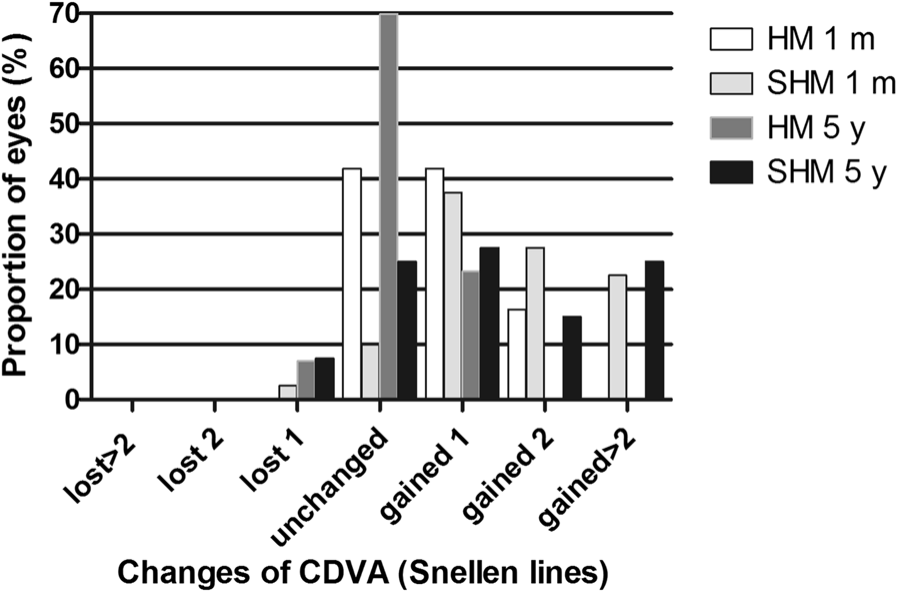
The percentage of eyes that gained/lost lines of CDVA at different time points of follow-up after EVO implantable collamer lens implantation between high myopia (HM) and super high myopia (SHM) groups. m, month; y, year

As shown in Fig. 1, the safety index (postoperative CDVA/preoperative CDVA) of HM and SHM groups were 1.16 ± 0.16 and 1.39 ± 0.37 at 1 month postoperatively and 1.03 ± 0.10 and 1.32 ± 0.39 at 5 years postoperatively. There was significant difference between the two groups (*P* < 0.05). The preoperative, 1 month and 5 years postoperative CDVA were − 0.02 ± 0.04, − 0.08 ± 0.05, and − 0.03 ± 0.05 logMAR in the HM group (*P* < 0.05) and 0.12 ± 0.14, − 0.01 ± 0.10, and 0.02 ± 0.08 logMAR in the SHM group (*P* < 0.05), respectively. At 5 years postoperatively, 6.98% of eyes lost one line of CDVA, 23.26% of eyes gained one line, and 69.77% of eyes did not change compared to the baseline in the HM group, and 7.50% of eyes lost one line of CDVA, 27.50% of eyes gained one line, 15.00% of eyes gained two lines, 25.00% of eyes gained two or more lines, and 25.00% of eyes did not change compared to the baseline in the SHM group. The percentage of eyes with CDVA 20/20 or better at baseline and 1 month and 5 years postoperatively were 95.35%, 100.00% and 100.00% in the HM group and 40.00%, 75.00%, and 67.50% in the SHM group. All eyes in the HM group had CDVA 20/40 or better at all time points; while 90.00% of eyes in the SHM group had CDVA 20/40 or better at baseline, this percentage increased to 100.00% at both postoperative time points.

### Efficacy

**Fig. 2:**
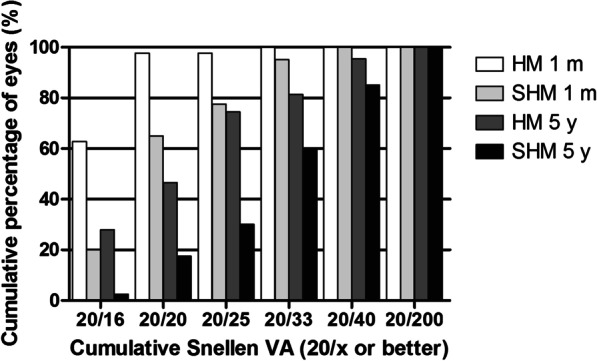
The cumulative percentage of UDVA at different time points after EVO implantable collamer lens implantation between high myopia (HM) and super high myopia (SHM) groups. m, month; y, year

As shown in Fig. 2, the efficacy index (postoperative UDVA/preoperative CDVA) of the HM group and SHM group were 1.10 ± 0.18 and 1.22 ± 0.33 at 1 month postoperatively and 0.83 ± 0.25 and 0.86 ± 0.32 at 5 years postoperatively, respectively. A significant difference at 1 month (*P* < 0.05) and no significant difference at 5 years postoperatively (*P* > 0.05) were observed between the two groups. The preoperative, 1 month and 5 years postoperative UDVA were 1.46 ± 0.42, − 0.06 ± 0.07, and 0.08 ± 0.15 logMAR in the HM group (*P* < 0.05) and 1.78 ± 0.34, 0.05 ± 0.12, and 0.22 ± 0.15 logMAR in the SHM group (*P* < 0.05), respectively. The percentages of eyes with UDVA 20/20 or better at 1 month and 5 years postoperatively were 97.67% and 46.51% in the HM group and 65.00% and 17.50% in the SHM group. The percentages of eyes with UDVA 20/40 or better were 100.00% and 95.35% in the HM group, 100.00% and 85.00% in the SHM group.

### Predictability

**Fig. 3:**
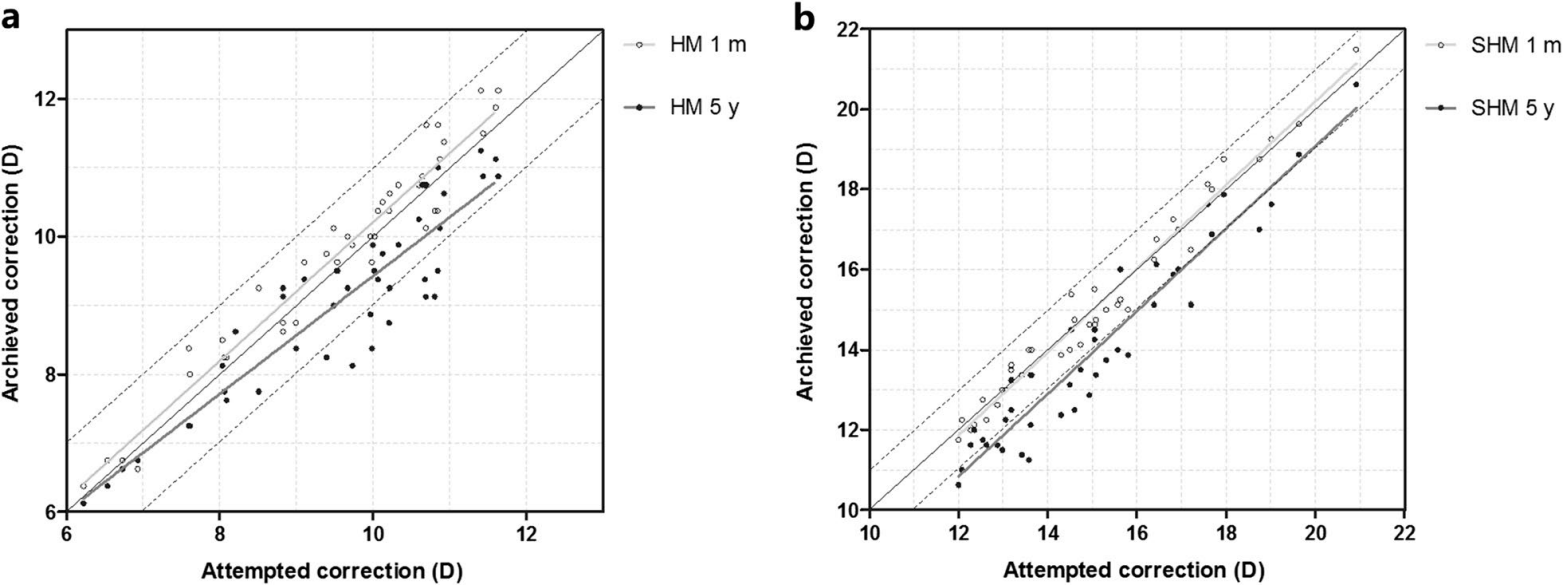
Scatter plot of attempted versus achieved correction (spherical equivalent) after EVO implantable collamer lens implantation between high myopia (HM) (**a**) and super high myopia (SHM) (**b**) groups. The black solid line represents achieved correction = attempted correction, the black dotted line represents achieved correction = attempted correction ± 1.00 D. m, month; y, year

A scatter plot with a best-fit line of the attempted versus the achieved spherical equivalent correction is shown in Fig. 3. At 1 month after surgery, the best mimic curve of the HM group was y = 1.01x + 0.16, with 81.40% eyes within ± 0.50 D and 100.00% eyes within ± 1.00 D of attempted correction value. The best mimic curve of the SHM group was y = 1.04x − 0.64, with 82.50% eyes within ± 0.50 D and 100.00% eyes within ± 1.00 D of attempted correction values. No significant differences were observed between the two groups (*P* > 0.05). At 5 years after surgery, the best mimic curve of the HM group was y = 0.85x + 0.88, with 60.47% eyes within ± 0.50 D and 79.07% eyes within ± 1.00 D of attempted correction values. Moreover, the best mimic curve of the SHM group was y = 1.03x − 1.54, with 22.50% eyes within ± 0.50 D and 47.50% eyes within ± 1.00 D of attempted correction values. There were significant differences between the two groups (*P* < 0.001).

### Changes of SE and axial length

**Fig. 4:**
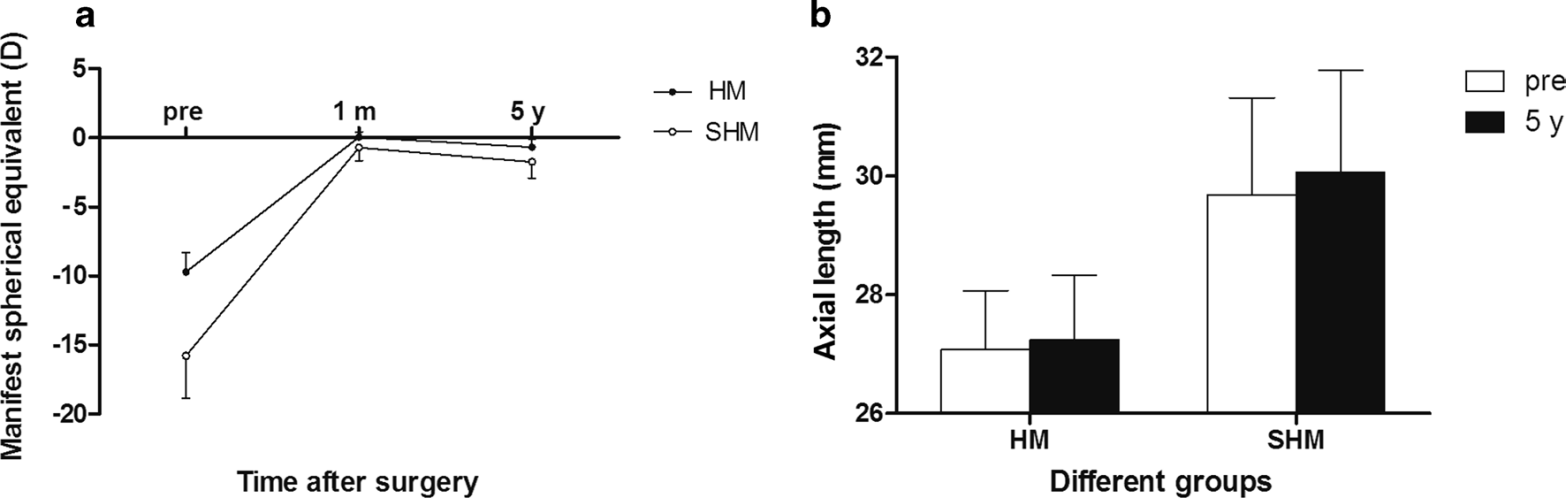
Manifest spherical equivalent (**a**) and axial length (**b**) between high myopia (HM) and super high myopia (SHM) groups after implantation of EVO implantable collamer lens over time. m, month; y, year; pre, preoperative

The SE of the HM group decreased from − 9.72 ± 1.41 D preoperatively to 0.04 ± 0.39 D at 1 month and − 0.67 ± 0.57 D at 5 years postoperatively and from − 15.78 ± 3.06 D preoperatively to − 0.69 ± 0.97 D at 1 month and − 1.74 ± 1.19 D at 5 years postoperatively in the SHM group (Fig. 4a). The differences in SE from 1 month to 5 years postoperatively for the HM and SHM groups were − 0.72 ± 0.54 D and − 1.05 ± 0.61 D, respectively (*P* < 0.05). The preoperative and 5 year postoperative axial lengths of the HM group were 27.08 ± 0.99 mm and 27.24 ± 1.09 mm, respectively, increasing by 0.16 ± 0.21 mm. The preoperative and last visit axial lengths of the SHM group were 29.68 ± 1.63 mm and 30.07 ± 1.72 mm, respectively, increasing by 0.39 ± 0.29 mm (Fig. 4b). There was a significant difference in axial length growth between the two groups (*P* < 0.001). The differences in SE were related to the axial length growth (*P* < 0.001).

### Intraocular pressure, vault, and endothelial cell density

**Fig. 5:**
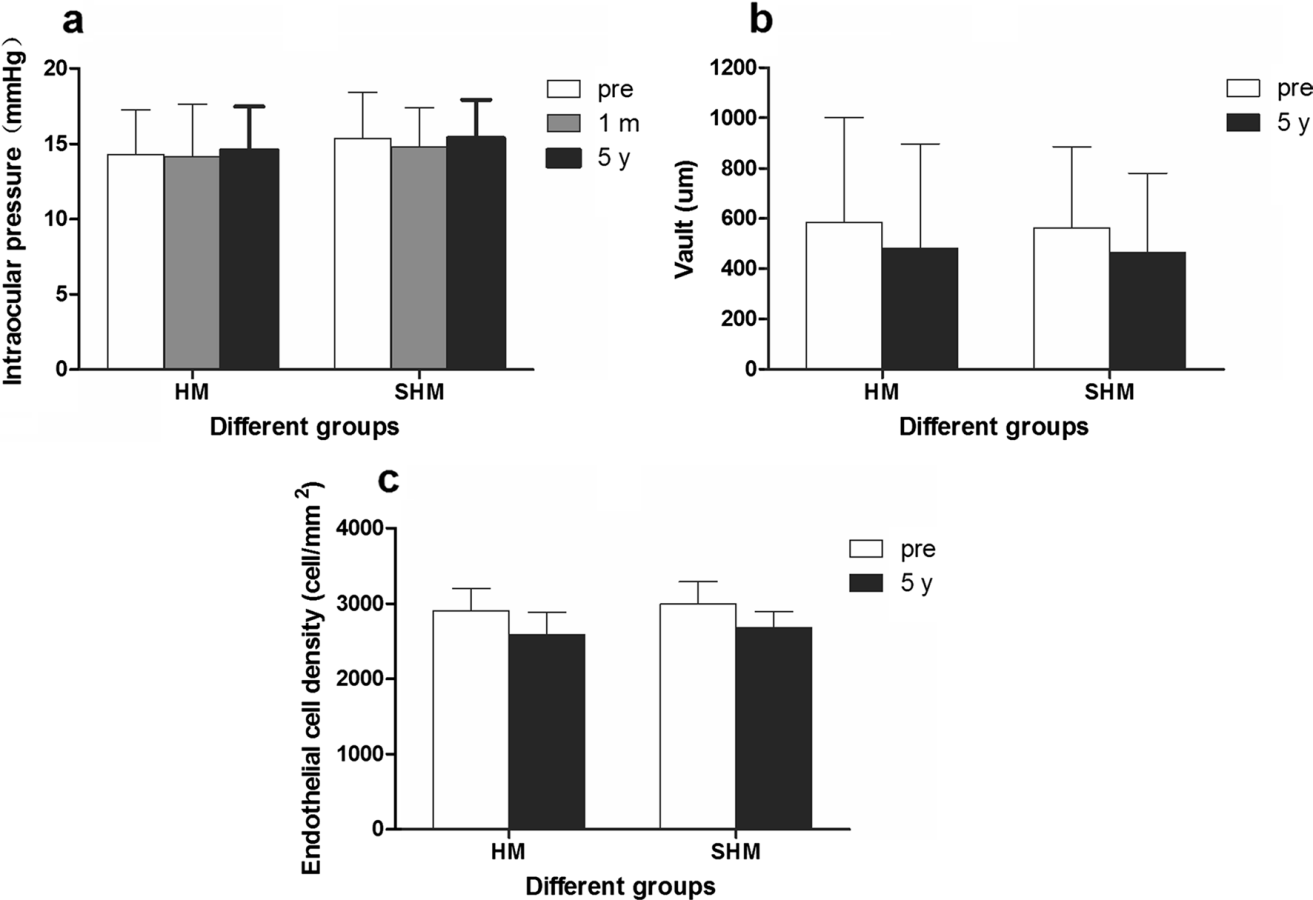
Intraocular pressure (**a**), vault (**b**) and the endothelial cell density (**c**) between high myopia (HM) and super high myopia (SHM) groups during the period of follow-up after implantation of EVO implantable collamer lens. m, month; y, year; pre, preoperative

The preoperative, 1 month and 5 years postoperative IOP of the HM group were 14.27 ± 2.99 (8.3–22.3) mmHg, 14.15 ± 3.47 (8.6–23.1) mmHg, and 14.58 ± 2.91 (11.5–24.5) mmHg, respectively, and these were 15.37 ± 3.05 (10.4–21.3) mmHg, 14.81 ± 2.61 (9.9–21.9) mmHg, and 15.40 ± 2.52 (10.6–20.0) mmHg, respectively, in the SHM group. There was no significant difference in IOP between the two groups at the three time points (*P* > 0.05; Fig. 5a).

The 1 month and 5 years postoperative vault of the HM group were 585.81 ± 416.53 (80–1680) μm and 481.16 ± 415.46 (0–1490) μm, respectively, decreasing by 104.65 ± 169.32 μm. The 1 month and 5 year postoperative vault of the SHM group were 563.75 ± 323.16 (130–1420) μm and 464.50 ± 316.31 (0–1300) μm, respectively, decreasing by 99.25 ± 176.77 μm. There was no significant difference in vault between the two groups at the two time points after surgery (*P* > 0.05). There was no significant difference in the reduction of vault between the two groups (*P* > 0.05; Fig. 5b).

The preoperative and 5 years postoperative endothelial cell densities of the HM group were 2689.56 ± 286.37 (2092–3213)/mm^2^ and 2591.03 ± 293.43 (1978–3125)/mm^2^, respectively, and the mean loss rate was 3.66%. The preoperative and 5-year postoperative endothelial cell densities of the SHM group were 2773.89 ± 206.10 (2411–3214)/mm^2^ and 2685.11 ± 213.30 (2305–3109)/mm^2^, respectively, and the mean loss rate was 3.20%. There was no significant difference in endothelial cell densities between the two groups before and 5 years after surgery (*P* > 0.05; Fig. 5c).

### Questionnaire results

From Table 2, there was no significant difference in scores between the two groups for any of the questions. It was observed that most HM patients of both groups were more satisfied with their images and more confident with themselves after EVO ICL implantation than before operation. About half of the HM patients thought that the ICL implantation was helpful to their careers. All patients admitted that the ICL implantation had brought convenience to their lives. In particular, the proportion of these changes in patients with SHM was higher.

**Table 2:** Distribution of postoperative questionnaire

Question	HM group	SHM group	*P* value
Q1	87.50%	90.91%	0.711
Q2	87.50%	90.91%	0.711
Q3	45.83%	59.09%	0.369
Q4	100.00%	100.00%	1.000
Q5	91.67%	100.00%	0.116
Q6	87.50%	95.45%	0.339

*HM* high myopia; *SHM* super high myopia; *Q *question

Results are expressed as a percentage of the positive answer

## Discussion

EVO ICL implantation is a good choice for patients with HM. In this study, the subjects were divided into HM and SHM groups according to SE. UDVA, CDVA, SE, IOP, axial length, endothelial cell density, and vault were measured before and after surgery to evaluate the long-term safety, efficacy, and stability of EVO ICL implantation in the correction of different degrees of HM.

Our study demonstrated that EVO ICL implantation has good long-term safety and efficacy in the correction of HM and SHM. The safety indices of the HM and SHM groups were all above 1.00, from 1 month to 5 years after surgery. The postoperative UDVA and CDVA of the SHM group were worse than those of the HM group, which may be due to the poor preoperative UDVA and CDVA, as well as the severe myopia in the SHM group before surgery. However, the CDVA of the SHM group was more likely to improve because the retinal imaging magnification was higher than that of the HM group. One month after surgery, the efficacy indices of the HM and SHM groups were all above 1.00. However, the efficacy index of the two groups decreased and was lower than 1.00 at 5 years after surgery. The decrease in UDVA and the efficacy index were associated with the refractive change and the axial length change at 5 years after surgery.

Both the HM and SHM groups showed good predictability 1 month after surgery. The predictability results at 5 years after surgery showed a tendency of under-correction, and the degree of under-correction was more obvious in the SHM group than in the HM group, which was caused by myopic drift, and myopic progression was more severe in SHM. The SE of the SHM group was higher than that of the HM group at 1 month and 5 years postoperatively because some patients had residual SE as their SE exceeded the upper limit of ICL correction. The progression of SE in the HM group and SHM group were − 0.72 ± 0.54 D and − 1.05 ± 0.61 D, respectively, and the mean axial length growth were 0.16 ± 0.21 mm and 0.39 ± 0.29 mm, respectively. There was a correlation between the progression of myopia and axial length growth. Our study showed that the SHM group had more progression of myopia and increased axial length growth. In Kimiya's [16] yearlong study, after the implantation of EVO ICL, the SE progression of low myopia group (mean SE − 4.29 ± 1.31 D) and HM group (mean SE − 10.13 ± 2.64 D) were − 0.12 ± 0.34 D and − 0.18 ± 0.43 D, respectively, suggesting that the higher the degree of myopia, the more it progresses. The change in postoperative SE is related to preoperative SE [17]. Therefore, patients with HM, especially those with SHM, should be explained prior to surgery that ICL implantation cannot guarantee the stability of myopia, and there is still the possibility of progression of myopia after surgery. HM, in particular SHM, is basically axial myopia (a myopic refractive state primarily resulting from a greater than normal axial length) [18]. Once the axial length increases, it will lead to the progression of myopia and the decrease of UDVA.

IOP after ICL implantation has also been an area of focus. There was no postoperative IOP increase in either group, indicating that EVO ICL can maintain stable IOP without preoperative iridotomy. Moreover, EVO ICL implantation eliminates the need for Nd:YAG laser iridotomy and reduces the IOP increase caused by pigment dissemination [19, 20]. In our study, the vault of the two groups showed a decreasing trend in the long-term follow-up of 5 years after surgery, and there was no statistical difference between the two groups. The rate of decrease of vault in the two groups was about 20 μm/year, which was similar to those reported in previous studies [21–24]; the vault and its change were independent of the preoperative SE. The mean 5-year endothelial cell loss rates in the HM and SHM groups were 3.66% and 3.20%, respectively. There was no significant difference between the two groups, which was in line with the physiological endothelial cell loss rule. However, there have been reports of large differences in the rate of endothelial loss [23–27]. The new central port design of the EVO ICL changes the aqueous flow and may therefore influence corneal endothelium cells [28], suggesting that the presence of a central hole does not increase the long-term loss of endothelial cells.

There were no significant differences in the questionnaire scores between the two groups. It can be seen from the results that ICL implantation not only brings a visual correction effect but also improves patients' lives and careers. Most of the patients had better self-images, more confident personalities, and smoother careers than before. For patients with HM and SHM, the value of ICL implantation is not only reflected in visual correction, but also in life.

This study has some limitations, which include the relatively small sample size from a statistical standpoint, lack of a low myopia array, and lack of comparison of visual quality. Besides, this prospective study only had three data points and the rate of missed follow-up in the intermediate period was high because of the Covid-19 situation. This study focused on the 5-year results after surgery, therefore, the data in the intermediate period was not included.

## Conclusions

In summary, our results suggest that EVO ICL implantation is safe, effective, and predictable for correcting HM and SHM. CDVA improved after surgery for the SHM group, but the growth of axial length still needs attention.

## Data Availability

Not applicable.
